# AP-1 Is a Component of the Transcriptional Network Regulated by GSK-3 in Quiescent Cells

**DOI:** 10.1371/journal.pone.0020150

**Published:** 2011-05-25

**Authors:** John W. Tullai, Silvia Tacheva, Laura J. Owens, Julie R. Graham, Geoffrey M. Cooper

**Affiliations:** 1 Department of Biology, Boston University, Boston, Massachusetts, United States of America; 2 Program in Molecular Biology, Cell Biology and Biochemistry, Boston University, Boston, Massachusetts, United States of America; Texas A&M University, United States of America

## Abstract

**Background:**

The protein kinase GSK-3 is constitutively active in quiescent cells in the absence of growth factor signaling. Previously, we identified a set of genes that required GSK-3 to maintain their repression during quiescence. Computational analysis of the upstream sequences of these genes predicted transcription factor binding sites for CREB, NFκB and AP-1. In our previous work, contributions of CREB and NFκB were examined. In the current study, the AP-1 component of the signaling network in quiescent cells was explored.

**Methodology/Principal Findings:**

Using chromatin immunoprecipitation analysis, two AP-1 family members, c-Jun and JunD, bound to predicted upstream regulatory sequences in 8 of the 12 GSK-3-regulated genes. c-Jun was phosphorylated on threonine 239 by GSK-3 in quiescent cells, consistent with previous studies demonstrating inhibition of c-Jun by GSK-3. Inhibition of GSK-3 attenuated this phosphorylation, resulting in the stabilization of c-Jun. The association of c-Jun with its target sequences was increased by growth factor stimulation as well as by direct GSK-3 inhibition. The physiological role for c-Jun was also confirmed by siRNA inhibition of gene induction.

**Conclusions/Significance:**

These results indicate that inhibition of c-Jun by GSK-3 contributes to the repression of growth factor-inducible genes in quiescent cells. Together, AP-1, CREB and NFκB form an integrated transcriptional network that is largely responsible for maintaining repression of target genes downstream of GSK-3 signaling.

## Introduction

The serine/threonine kinase glycogen synthase kinase-3 (GSK-3) is a master regulator of a variety of cellular processes. First characterized as the kinase responsible for phosphorylating and inactivating glycogen synthase, GSK-3 now has recognized roles in controlling cell proliferation, survival and differentiation. Abnormal GSK-3 regulation has been associated with many human diseases including diabetes, heart disease, cancer, Alzheimer's disease and schizophrenia [1], [2], [3].

GSK-3 has two widely expressed mammalian isoforms, GSK-3α and GSK-3β, both of which are subject to regulation by the PI 3-kinase/Akt pathway [4]. GSK-3 has been shown to regulate cell survival and proliferation downstream of PI 3-kinase signaling through phosphorylation of cyclin D1 [5], Mcl-1 [6], and eukaryotic translation initiation factor 2B (eIF2B) [7], [8], as well as a variety of transcription factors [1], [3]. GSK-3 is also regulated through the Wnt pathway. Wnt signaling results in a decrease in the phosphorylation of β-catenin by GSK-3, causing a corresponding increase in the transcriptional activation of β-catenin/TCF target genes [9].

Unlike most protein kinases, GSK-3 is constitutively active in quiescent cells, and undergoes an inhibitory phosphorylation by Akt (on serine 9 for GSK-3β, and on serine 21 for GSK-3α) in the presence of growth factors [4]. The activity of GSK-3 in quiescent cells suggests that it may actively maintain repression of growth factor-regulated genes in the absence of PI 3-kinase signaling. We have investigated the role of GSK-3 in quiescence by combining global expression profiling and computational analyses to examine gene expression downstream of PI 3-kinase/Akt/GSK-3 signaling [10]. These studies identified a set of twelve immediate early genes whose induction following growth factor stimulation of quiescent T98G human glioblastoma cells was dependent upon PI 3-kinase and which could also be induced by direct inhibition of GSK-3 without growth factor stimulation [10], [11]. These genes mainly encoded growth factors and transcription factors involved in cell proliferation, so their repression by GSK-3 presumably contributed to maintenance of the quiescent state of the cell.

The identification of a set of genes that required GSK-3 to maintain their repression during quiescence allowed us to investigate the transcriptional network downstream of GSK-3 signaling. Since the expression of co-regulated genes may be mediated by common transcription factors, we examined the upstream sequences of the twelve GSK-3 repressed genes to identify statistically over-represented and evolutionarily conserved transcription factor binding sites. This computational analysis predicted AP-1, as well as CREB and NFκB transcription factors, as potential regulators of these genes downstream of GSK-3 [10], [12].

In the present study, we have investigated the role of AP-1 family members in GSK-3 mediated transcriptional regulation. Two AP-1 family members, c-Jun and JunD, bound to predicted upstream regulatory sequences in 8 of the 12 GSK-3-regulated genes. Consistent with previous studies demonstrating inhibition of c-Jun by GSK-3 [13], [14], c-Jun was phosphorylated by GSK-3 in quiescent cells. The association of c-Jun with its target sequences was increased by growth factor stimulation as well as by GSK-3 inhibition, and a physiological role for c-Jun was demonstrated by siRNA inhibition of gene induction. These results indicate that inhibition of c-Jun by GSK-3 contributes to the repression of growth factor-regulated genes during quiescence. Moreover, together with previous studies, these findings delineate an integrated transcriptional network in which AP-1, CREB and NFκB play major roles in GSK-3-mediated repression of target genes in quiescent cells.

## Results and Discussion

### Binding of c-Jun to GSK-3 Target Genes

**Figure 1:**
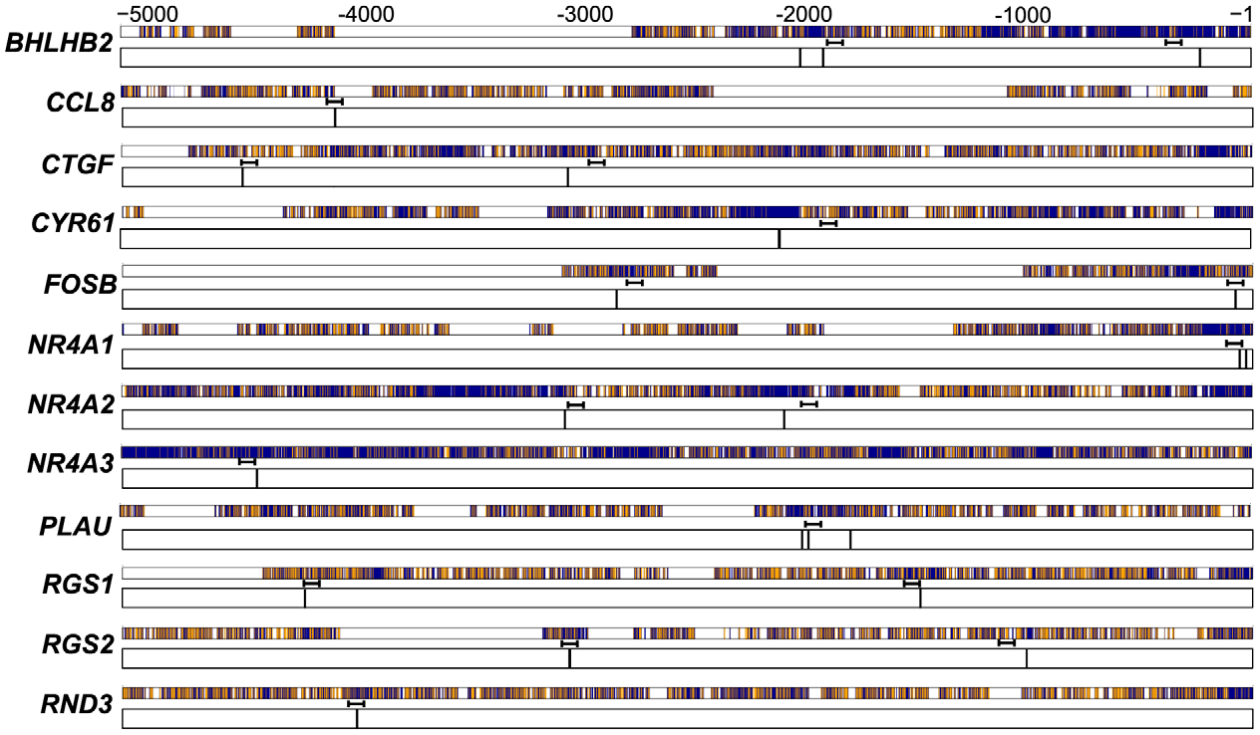
Conserved AP-1 sites predicted in the upstream regions of GSK-3 regulated genes. Predicted AP-1 binding sites are indicated by vertical black lines in the 5-kb aligned sequences of human and mouse genes [10]. Numeric positions are relative to the transcription start site. Positions of the ChIP PCR amplicons are indicated. Refer to Table S1 for precise positions of predicted sites and of primers. Alignments are indicated as dark blue, match; orange, mismatch; white, aligned to gap.

**Figure 2:**
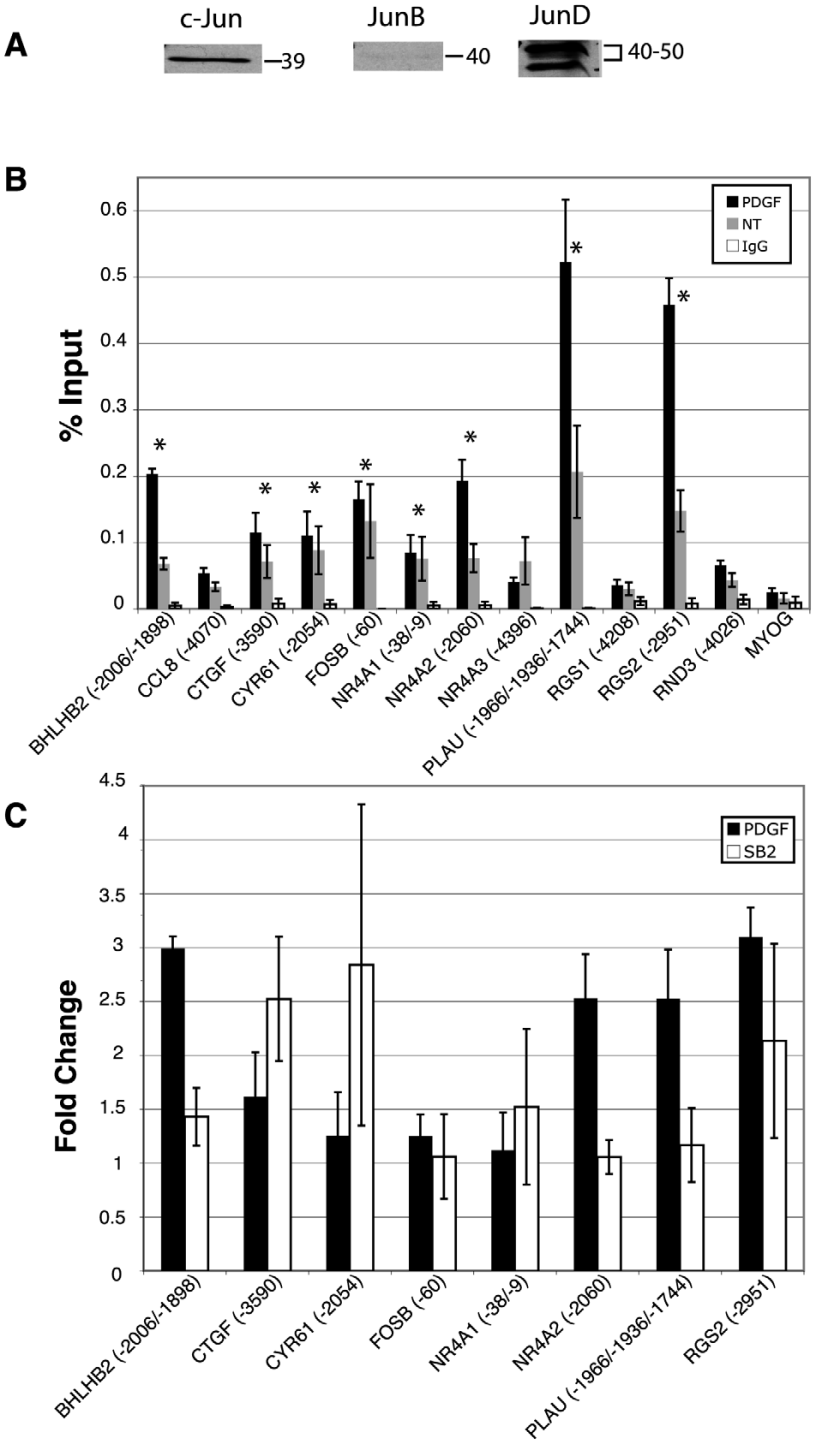
Analysis of c-Jun binding and recruitment to predicted AP-1 sites by chromatin immunoprecipitation. A. Quiescent T98G cell extracts were immunoblotted with anti-cJun, anti-JunB and anti-JunD antibodies. Images were taken from different parts of the same autoradiography film, and therefore have identical exposures. Sizes indicated are in kDa. As expected, JunD has two protein products which run as a 40–50 kDa doublet B. Quiescent T98G cells were treated with PDGF for 30 minutes or left untreated (NT), and then chromatin was immunoprecipitated with anti-c-Jun antibody or normal rabbit IgG. Only the PDGF-stimulated samples are plotted for the normal rabbit IgG immunoprecipitates. The numbers in parentheses refer to the 5′-most position of the putative AP-1 binding site (see Figure 1) relative to the transcription start site. When more than one position is listed, this refers to all possible sites that would be detected within the resolution limits of the ChIP PCR amplicon (approximately ±250 nucleotides). For those genes with multiple putative AP-1 sites, only one representative site (if all were negative for a given gene) or that site which indicated binding are shown for clarity. For a complete list of all tested sites, see Table S1. Data are presented as percent input averaged from 4 separate experiments ± S.E. *MYOG* served as a negative control promoter. Asterisks indicate greater than 3-fold binding as compared to *MYOG* in both untreated and PDGF treated samples C. Recruitment of c-Jun upon stimulation with PDGF or direct inhibition of GSK-3 with SB-216763. Quiescent T98G cells were treated with PDGF for 30 minutes or left untreated, or treated with SB-216763 for 1 hour or with DMSO vehicle control. Only those genes that initially showed c-Jun binding (panel A) are shown. Data are presented as fold change over untreated (for PDGF) or fold change over DMSO vehicle control (for SB-216763), and are averaged from 4 separate experiments ±S.E. No significant change was observed in the normal rabbit IgG or *MYOG* samples (not shown).

Our previous computational analysis identified a total of 22 conserved, putative AP-1 sites across all 12 of the genes that were induced by inhibition of GSK-3 (Figure 1). The AP-1 family of transcription factors is made up of three Jun family members (c-Jun, JunB, and JunD) and four Fos family members (c-Fos, FosB, Fra-1 and Fra-2). AP-1 is a dimeric transcription complex that is formed from Jun-Jun family homodimers, or Jun-Fos family heterodimers [15], [16]. It is the particular combination of the dimers that determines the transcriptional activity. FosB, c-Fos, and c-Jun are activators, whereas JunD, JunB, Fra-1 and Fra-2 have weaker transactivation domains and can act as repressors by competing for c-Fos, FosB and c-Jun binding. To determine which family members are present in quiescent T98G cells, immunoblots for all members were conducted. As expected, c-Fos, FosB, Fra-1 and Fra-2 were not detected in the quiescent cells, as they require growth factor signaling for their induction [17], [18], [19], [20] (data not shown). c-Jun, JunD and JunB were all detected by immunoblots, although JunB was only weakly detected (Figure 2A). c-Jun, JunD and JunB therefore were pursued in subsequent chromatin immunoprecipitation (ChIP) experiments.

We sought to determine if c-Jun was bound to these predicted sites by ChIP analysis of quiescent T98G cells, as well as following stimulation with platelet-derived growth factor (PDGF). T98G cells were rendered quiescent as described in Materials and Methods, and then stimulated for 30 minutes with PDGF, which was the growth factor used in the initial studies of the PI 3-kinase/Akt/GSK-3 regulated genes [11], from which the GSK-3 regulated subset was derived [10]. Eight of 12 genes (*BHLHB2*, *CTGF*, *CYR61*, *FOSB*, *NR4A1*, *NR4A2*, *PLAU*, and *RGS2*) demonstrated occupancy/binding by c-Jun (greater than 3-fold as compared to the negative control promoter, *MYOG*) in both untreated and PDGF treated samples (Figure 2B). PDGF treatment resulted in a greater than 2-fold increase in binding of c-Jun at 4 sites in 4 different genes (*BHLHB2*, *NR4A2*, *PLAU* and *RGS2*) as well as a moderate increase in binding (1.6 fold) for *CTGF* (Figure 2B and C). We next tested for binding and recruitment of c-Jun following direct inhibition of GSK-3 with the small molecule inhibitor SB-216763 in the absence of growth factor stimulation. Interestingly, inhibition of GSK-3 also resulted in a greater than 2-fold increase in binding of c-Jun to the upstream sites in *CTGF*, *CYR61* and *RGS2* (Figure 2C), indicating a direct effect of GSK-3 on c-Jun. Overall, the predicted upstream sequences of 8 out of 12 genes were bound by c-Jun and the occupancy of c-Jun at 6 of these genes was increased following either growth factor stimulation or direct inhibition of GSK-3.

### Binding of JunD to GSK-3 Target Genes

**Figure 3:**
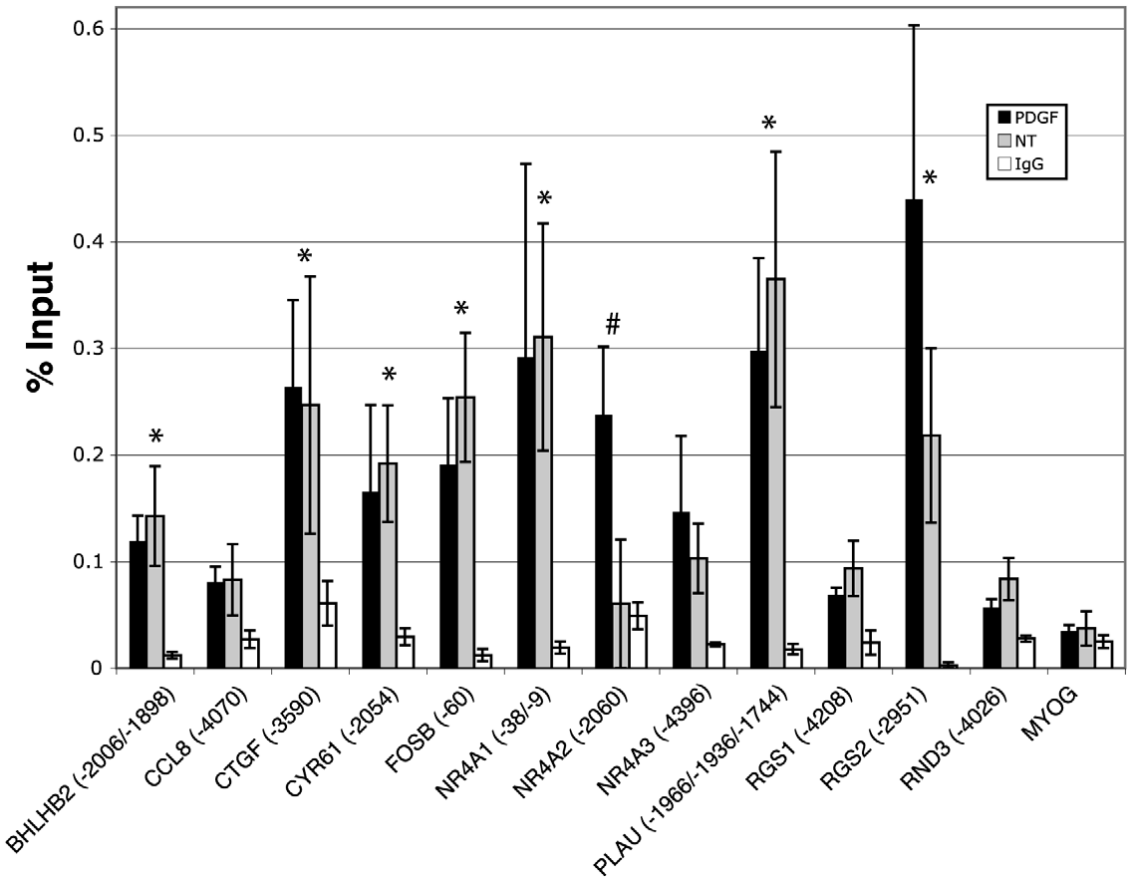
Analysis of JunD binding to predicted AP-1 sites by chromatin immunoprecipitation. Quiescent T98G cells were treated with PDGF for 30 minutes or left untreated (NT), and then chromatin was immunoprecipitated with an anti-JunD antibody or normal rabbit IgG. Only the PDGF-stimulated samples are plotted for normal rabbit IgG. The numbers in parentheses refer to the 5′-most position of the putative AP-1 binding site (see Figure 1) relative to the transcription start site. Data are presented as percent input averaged from 4 separate experiments ± S.E. *MYOG* served as a negative control promoter. * indicates greater than 3-fold binding compared to *MYOG* in both untreated and PDGF treated samples; # indicates greater than 3-fold binding compared to *MYOG* in the PDGF treated sample only.

JunD and JunB AP-1 family members have weaker activation domains and can potentially act as repressors that antagonize or inhibit the binding of activating family members [15], [16]. We therefore hypothesized that gene induction through AP-1 may be the result of JunD and/or JunB being displaced by an activating family member such as c-Jun. To test this, we conducted ChIP analysis for both JunB and JunD at the predicted AP-1 sites illustrated in Figure 1. JunB ChIP experiments did not show binding or recruitment greater than that of the negative control promoter, *MYOG*, or than that of the IgG control (data not shown). In contrast, ChIP analysis for JunD indicated that for many of the genes, JunD was bound to the upstream regions (Figure 3). In all, 8 out of 12 genes showed JunD binding greater than 3-fold as compared to the negative control promoter, *MYOG*. For seven of the genes showing JunD binding (*BHLHB2*, *CTGF*, *CYR61*, *FOSB*, *NR4A1*, *PLAU*, and *RGS2*), the relative binding of the PDGF and untreated (NT) samples were not significantly different, but both higher than that of the negative control, *MYOG*, and of the control IgG sample. The eighth gene, *NR4A2*, showed increased binding of JunD upon PDGF stimulation. Binding of JunD to these 8 genes corresponded to 100% overlap with those sites to which we demonstrated c-Jun binding (see Figure 2B).

These results suggested that JunD was occupying the same AP-1 sites as c-Jun, but with the exception of *NR4A2*, was not changing in response to growth factor stimulation. This may reflect the presence of c-Jun-JunD heterodimers at some or all of these AP-1 binding sites. Genes targeted by AP-1 may therefore be activated by recruitment of the activating partner, c-Jun, or by posttranslational modifications of the complex to stimulate target gene expression.

### Inhibition of GSK-3 Leads to Dephosphorylation and Stabilization of c-Jun

**Figure 4:**
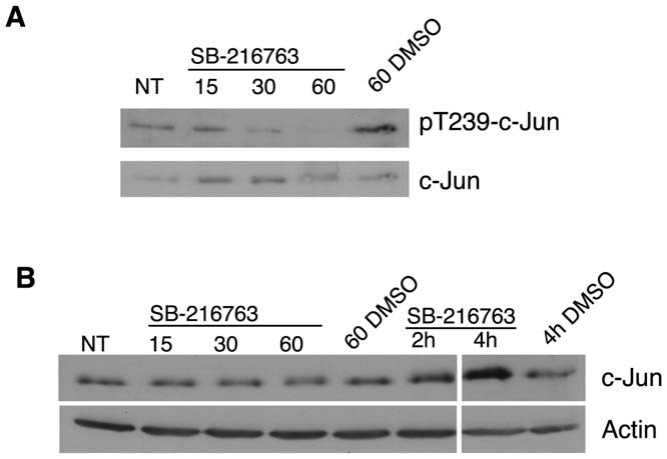
Effect of GSK-3 inhibition on c-Jun phosphorylation and stability. T98G cells were rendered quiescent by serum starvation for 72 hours. Cells were then left untreated (NT), or treated with SB-216763 or vehicle control (DMSO) for the times indicated. Cell extracts were immunoblotted in parallel with anti-phospho threonine 239-c-Jun, pan-c-Jun and β-actin antibodies. Data shown are representative of three separate experiments. The left and right panels of the immunoblot in (B) were taken from the same autoradiography film, and therefore are identical exposure times.

Since the recruitment of c-Jun to its target sites was stimulated by inhibition of GSK-3, we investigated the effect of GSK-3 inhibition on c-Jun phosphorylation. It has been previously shown that GSK-3 phosphorylates c-Jun on threonine 239 [21]. This phosphorylation event has been shown to block c-Jun DNA binding activity [13] and transactivation [22], and target it for ubiquitination and proteasomal degradation [14]. We therefore assessed the phosphorylation status of c-Jun with the use of phospho-specific antibodies (Figure 4A). Quiescent T98G cells were treated for a time course up to 60 minutes with the GSK-3 inhibitor SB-216763, or the corresponding vehicle control, without growth factor stimulation. This was the treatment time corresponding to the gene inductions described previously [10]. As expected, phosphorylation of c-Jun threonine 239 was readily detectable in quiescent cells, consistent with the increased kinase activity of GSK-3 [10], [12]. As compared to the untreated sample (NT), phosphorylation at threonine 239 decreased as rapidly as 15 minutes following addition of SB-216763, and declined for the duration of the time course. The 60-minute vehicle control was unchanged.

We next conducted a more extensive time course of GSK-3 inhibition to see if we could observe a stabilization of c-Jun (Figure 4B). Quiescent T98G cells were again treated with SB-216763 in the absence of growth factors. The levels of c-Jun remained relatively unchanged until 2 hours, when a modest increase in signal was observed as compared to the vehicle control. The 4-hour time point showed a more marked increase in signal as compared to the β-actin loading control, suggesting that the amount of c-Jun protein had increased during the duration of the experiment. This apparent increase in the c-Jun signal upon dephosphorylation of threonine 239 would be consistent with a stabilization of the protein previously described [14], and with the corresponding gene activation observed in the present study. Taken together, GSK-3 inhibition caused the rapid dephosphorylation of c-Jun threonine 239 (allowing DNA binding and transactivation activity), and subsequent stabilization of the protein.

### Effect of c-Jun siRNA on the Induction of GSK-3-regulated genes

**Figure 5:**
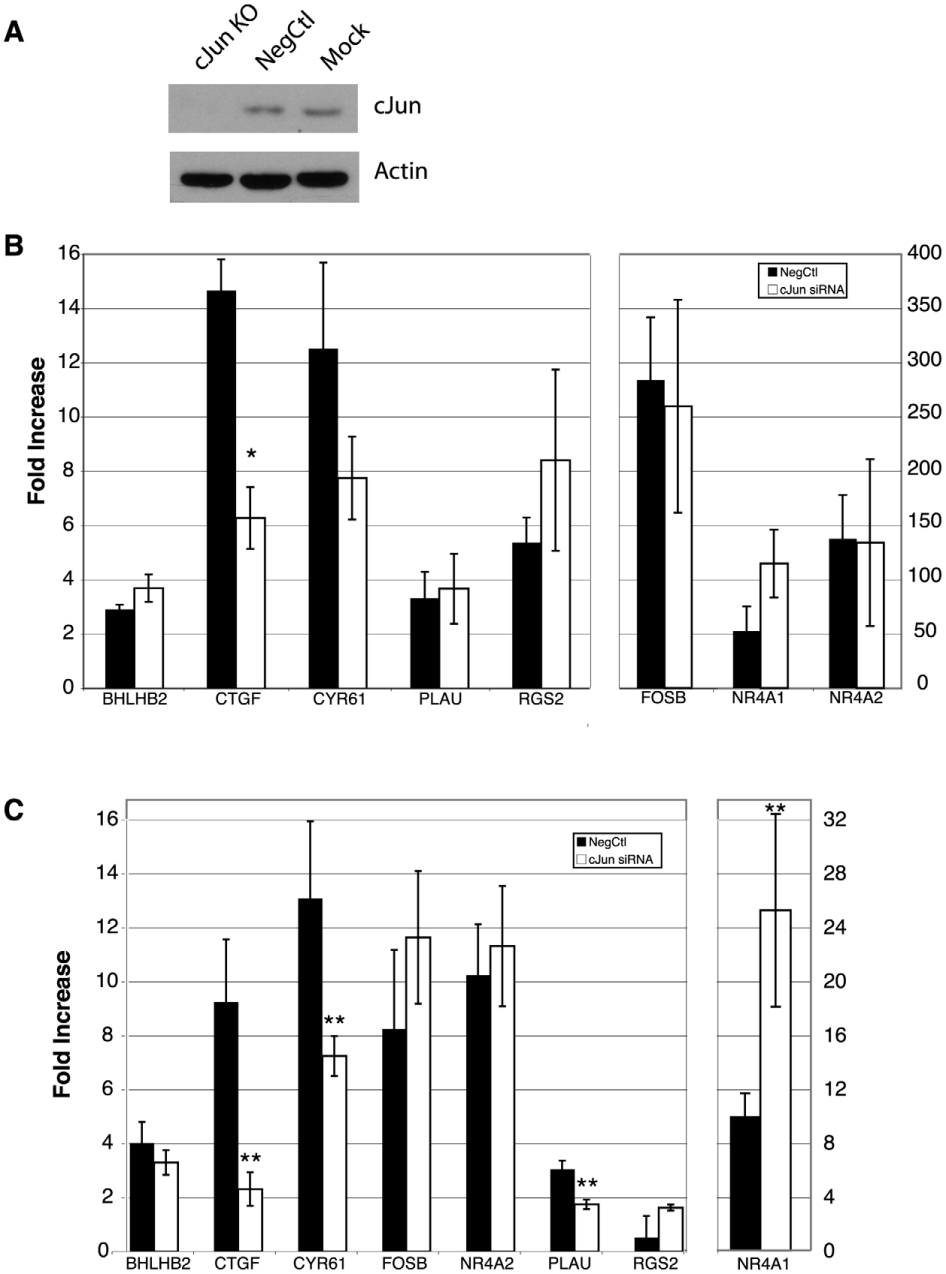
Effect of c-Jun siRNA on gene induction. A. Knockdown of c-Jun by siRNA. Cells were transfected with either the c-Jun siRNA or a nonspecific construct (NegCtl), or with no construct added (Mock) and then analyzed by c-Jun and β-actin immunoblots. B. Effect of c-Jun knockdown on PDGF induction. T98G cells were transfected with a c-Jun or nonspecific negative control siRNA for 24 hours, and then serum starved for 48 hours. Cells were then stimulated with PDGF for 30 minutes. Expression of the indicated genes was determined by realtime RT-PCR. Data are presented as fold-change as compared to untreated. Data are means for 4 separate experiments ± S.E. * *p*<0.01. C. Effect of c-Jun knockdown on SB-216763 induction. Transfection and starvation were performed as above, and then cells were treated for 1 hour with SB-216763. Data are presented as fold change as compared to vehicle control (DMSO). Data are means for 4 separate experiments ± S.E., **p<0.05.

To determine whether c-Jun is required for the induction of the GSK-3 regulated genes, RNA interference experiments were performed. Transfection of a specific c-Jun siRNA for 24 hours, followed by 48 hours of serum starvation resulted in a knockdown of greater than >90% in T98G cells (Figure 5A). At the end of the 48-hour serum starvation, the effect of the c-Jun knockdown was tested for both the PDGF and SB-216763 induction of the 8 genes for which we demonstrated c-Jun binding. Treatment with c-Jun siRNA decreased the induction of *CTGF* by PDGF greater than two-fold (*p*<0.01) (Figure 5B). The induction *CYR61* by PDGF was also decreased nearly two-fold in the presence of the c-Jun siRNA, but did not reach statistical significance (*p* = 0.11) (Figure 5B). *RGS2*, despite revealing the most dramatic c-Jun recruitment in the ChIP assays upon PDGF stimulation (Figure 2B), does not appear to require c-Jun for its PDGF-mediated induction. RNAi against c-Jun significantly blocked the induction of *CTGF*, *CYR61* and *PLAU* (*p*<0.05) resulting from inhibition of GSK-3 with SB-216763 (Figure 5C). These experiments directly demonstrated that c-Jun is required for the induction of these three genes following GSK-3 inhibition.

### Role of AP-1 in the Transcriptional Network Downstream of GSK-3

Previous studies showed that the GSK-3 regulated genes were also targeted by CREB [10] and NFκB [12]. We therefore sought to determine whether there were synergistic or antagonistic interactions between c-Jun, CREB and NFκB in the transcriptional response of these genes to inhibition of GSK-3. It is noteworthy in this regard that knockdown of c-Jun resulted in a greater than 2-fold increase in the induction of *NR4A1* upon inhibition of GSK-3 with SB-216763 (Figure 5C). Thus, rather than activating transcription of *NR4A1*, c-Jun appears to interfere with the induction of this gene, suggesting an inhibitory role for AP-1 upon *NR4A1* transcription. Importantly, *NR4A1* is also targeted by both CREB and NFκB, with siRNA knockdowns of either of these factors resulting in a significant inhibition of *NR4A1* induction [10], [12]. It is thus noteworthy that AP-1 and CREB bind to similar sites [23], and that the AP-1 binding sites at −9 and at −38 (relative to the transcription start site) were also found to be binding sites for CREB [10]. This suggests that c-Jun may suppress induction of *NR4A1* by sterically interfering with the ability of CREB to bind at these sites and activate transcription.

**Figure 6:**
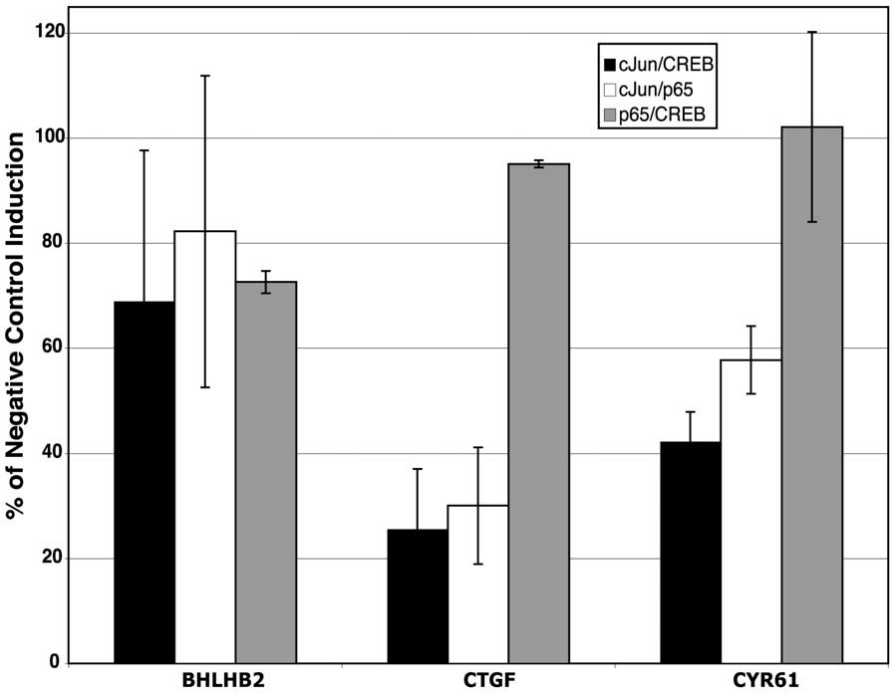
Effect of double siRNAs on gene induction by inhibition of GSK-3. Cells were rendered quiescent and transfected with the indicated combination of siRNAs. A total of 2.5 nM of each siRNA (5 nM total) was used as compared to 5 nM of negative control siRNA. Knockdown efficiencies of c-Jun, p65 and CREB were greater than 90% under these conditions. Data are presented as % of the SB-216763 induction as compared to the induction in the presence of the negative control. All data points are averages of a minimum of n = 3 (except for the p65/CREB combination, which is n = 2), ± S.E.

We investigated the possible synergistic roles of c-Jun, NFκB and CREB by performing double knockdown experiments with pairs of siRNAs for all 3 of these transcription factors. Overall, the pairwise knockout results were not significantly different from the effects of siRNA against the individual factors. Representative results are presented in Figure 6. For example, *BHLHB2* has upstream binding sites for c-Jun and NFκB [12], but induction of *BHLB2* was not significantly inhibited by siRNAs against either NFκB [12] or c-Jun (see Figure 5) individually. Likewise, induction of *BHLB2* was not significantly inhibited by any of the combinations of siRNAs against these factors (Figure 6).

*CTGF* and *CYR61* are examples of genes with c-Jun binding sites whose induction by inhibition of GSK-3 was inhibited by c-Jun siRNA (see Figure 5). Both of these genes have binding sites for NFκB and *CYR61* also has a binding site for CREB, but the induction of these genes in response to inhibition of GSK-3 was not significantly inhibited by either NFκB or CREB siRNAs [10], [12]. Consistent with the results of siRNAs against these individual factors, induction of these genes was inhibited only by combinations of siRNAs which included c-Jun siRNA, and the combinations had no greater effect than c-Jun siRNA alone (compare Figures 5 and 6). The triple knockdown of these factors likewise did not affect the SB-216763 induction of *BHLHB2*, *CTGF* or *CYR61* (data not shown) as compared to the knockdown of c-Jun alone.

**Figure 7:**
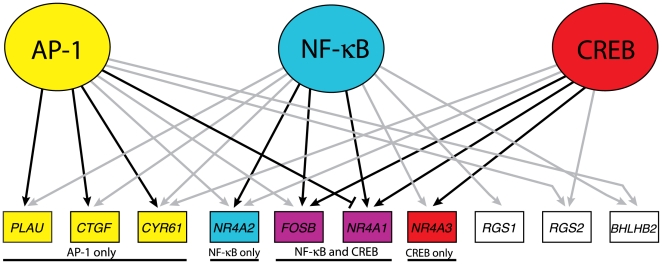
Model of the GSK-3 transcriptional network in quiescent cells. The AP-1 data has been combined with that of CREB [10] and NFκB [12]. Both grey and black arrows indicate ChIP binding by the given factor to the gene's upstream sequence. Black arrows indicate that siRNA against the factor blocked induction of the gene in response to SB-216763 treatment greater than two-fold and statistically significant (*p*<0.05). The blunt-ended edge between AP-1 and *NR4A1* indicates a greater than two-fold inhibition (*p*<0.05) of induction in the presence of AP-1. *RND3* and *CCL8* have been excluded from the illustration, as no ChIP binding nor transcription factor knockdown data indicated any functional connections with AP-1, NFκB or CREB.

The results of gene regulation downstream of GSK-3 by AP-1 are integrated with the results of our previous studies on CREB [10] and NFκB [12] in Figure 7. These 3 factors comprise a transcriptional network that maintains repression of growth factor-inducible genes in quiescent cells. Of 12 genes that were inducible by inhibition of GSK-3 in quiescent T98G cells, 10 were targeted by at least one of these three transcription factors. Moreover, 4 genes were targeted by all 3 transcription factors and 5 genes by 2 of the 3 factors.

The role of these transcription factors, as assessed by the effects of siRNA knockdowns, varied for the different target genes in the network. The induction of 3 of the 10 genes (*RGS1*, *RGS2* and *BHLBH2*) was not significantly affected by siRNAs against AP-1, CREB, or NFκB, either alone or in combination, suggesting that other transcription factors play a dominant role in regulation of these genes. Induction of 5 of the other 7 genes was significantly inhibited by siRNA against 1 of the transcription factors that bound to their upstream sequences, and induction of 2 genes (*FOSB* and *NR4A1*) was inhibited by siRNAs against both CREB and NFκB. This suggests that one or two transcription factors play dominant roles in the induction of different target genes. Interestingly, induction of *NR4A1* was antagonized by AP-1, presumably as a result of competition for CREB binding as noted above.

AP-1 [15], [16], NFκB [24], [25] and CREB [26], [27] are all known to play important roles in the induction of immediate-early genes in response to growth factor stimulation. Furthermore, previous studies have shown that AP-1 [13], [22], NFκB [12], [28], [29], [30] and CREB [10], [31], [32], [33], [34] all can be inhibited by GSK-3. Our results indicate that these 3 factors comprise a transcriptional network whose inhibition by GSK-3 plays an important role in maintaining repression of growth factor-inducible genes during quiescence.

## Materials and Methods

### Cell Culture and Treatments

T98G human glioblastoma cells were grown in Minimal Essential Medium (Invitrogen) containing 10% fetal bovine serum (Hyclone) and penicillin-streptomycin (Invitrogen). For inhibitor and growth factor experiments, cells were switched to serum free media for 72 hours to place the cells in G_0_ arrest [35]. Arrested cells were then stimulated with 50 ng/mL human PDGF-BB (Peprotech) for 30 minutes (or left untreated), or 5 µM SB-216763 (BioMol) or DMSO vehicle for 1 hour.

### Chromatin Immunoprecipitation

Chromatin immunoprecipitations were performed as previously described [10] using 5 µg of anti-c-Jun (Santa Cruz, sc-1696X ), 5 µg of anti-JunD (Santa Cruz, sc-74), or 5 µg of normal rabbit IgG as a control (Santa Cruz, sc-2027). Salmon sperm DNA/Protein A agarose bead slurry (50%) immunoprecipitates were successively washed in low salt, high salt, and lithium chloride buffers before being washed twice with 1× TE (10 mM Tris-HCl, 1 mM EDTA pH 8.0). Immunoprecipitated chromatin was quantified with real-time PCR (see Figure 1 for mapped amplicons and Table S1 for precise locations of sites, primers and primer sequences). *MYOG* was used as the negative control for all ChIPs.

### Immunoblots

Whole cell lysates were electrophoresed in 10% polyacrylamide gels and transferred to either nitrocellulose or PVDF membranes. Membranes were then incubated with either anti-JunD (Santa Cruz, sc-74), anti-JunB (Santa Cruz, sc-73), anti-phospho threonine 239-c-Jun (Santa Cruz, sc-101720), anti-c-Jun (Santa Cruz, sc-1696) or anti-β actin (Sigma). Immunoblots were then incubated with a horseradish peroxidase secondary antibody (Bio-Rad) followed by visualization using chemiluminescence and exposure to autoradiography film.

### Quantitative RT-PCR

Total RNA was isolated using Trizol (Invitrogen) as recommended by the manufacturer. RNA was used in quantitative real-time reverse transcription polymerase chain reactions (RT-PCR) as previously described [11], except using a minimum of 0.75 µg of total RNA in the reverse transcription reaction. PCR primers are as previously described [10].

### RNA interference

Transfections were performed using pre-designed siRNAs against p65 (Ambion, S11915), CREB (Ambion, 109994), c-Jun (Ambion, s7658), or a non-specific negative control (Ambion, 4390843). Shortly before transfection, 10^5^ cells/ml were seeded on 60 mm plates in 4 ml of media containing 10% fetal bovine serum. Transfection reactions containing 5 nM of siRNA, 20 µl of HiPerfect (Qiagen) and 100 µl of serum-free media were incubated for 10 min at room temperature and added drop-wise onto the cells. For the double knockdowns, 2.5 nM of c-Jun siRNA, 2.5 nM of p65 siRNA and 2.5 nM of CREB siRNA were used. Cells were incubated at 37°C, 5% CO_2_ for 24 hrs and then serum starved for 48 hrs to induce quiescence prior to treatment with PDGF-BB for 30 minutes or SB-2167623 for 1 hour. Starvation times of 48 hrs were used in these experiments to minimize toxicity from the transfection; gene inductions were comparable after either 48 or 72 hrs of starvation [10]. Quiescent cells were then appropriately treated, after which RNA was extracted and analyzed by real-time RT-PCR.

## Supporting Information

Table S1 **Chromatin Immunoprecipitation Primers for Quantitative Real-Time PCR.** Real-time PCR primer sequences and predicted AP-1 binding site positions are shown. The “Sites” column denotes multiple predicted binding sites in one gene where applicable. The “Sites Covered” column, in turn, refers to those sites that are too close to be distinguished from one another (typically <250 nucleotides away from each other, the approximate resolution of the ChIP experiment), and therefore both (or in some cases, three sites) are tested with one primer set. * = Experiments with these primer pairs were performed, but the data is not shown. Results were negative.
(XLS)
